# Composite Aerogels of Carbon Nanocellulose Fibers and Mixed-Valent Manganese Oxides as Renewable Supercapacitor Electrodes

**DOI:** 10.3390/polym11010129

**Published:** 2019-01-13

**Authors:** Xiaoyu Guo, Qi Zhang, Qing Li, Haipeng Yu, Yixing Liu

**Affiliations:** Key Laboratory of Bio-Based Material Science and Technology, Ministry of Education, Northeast Forestry University, Harbin 150040, China; guoxiaoyu666@163.com (X.G.); qzhang_2013@163.com (Q.Z.)

**Keywords:** nanocellulose, manganese oxide, aerogel, composite, supercapacitor electrode, electrochemistry

## Abstract

Bio-waste derived nanocelluloses show excellent mechanical flexibility and self-aggregated capability, which enable them to be good supporting substrates for the synthesis of electroactive materials. Herein, we present a facile route for fabricating composite aerogels consisting of carbonized nanocellulose fibers (CNF) and mixed-valent manganese oxide (MnO*_x_*), toward supercapacitor applications. Mixed solutions of nanocellulose and manganese acetate with different ratios were prepared and freeze-dried into hybrid aerogels. The hybrid aerogels were then transformed into CNF/MnO*_x_* composites by a calcination process. The CNF membranes served as porous carbon nano-reservoirs for MnO*_x_* and electrolyte. The CNF/MnO*_x_* composites also kept a 3D porous aerogel structure with hierarchical pores, which enabled stable transport of both electrolyte ions and electrons to the electrode surface, leading to low a charge-transfer impedance and good electrochemical kinetics. The CNF/MnO*_x_*-based symmetric supercapacitor showed a satisfied energy density and power density of 37.5 Wh kg^−1^ and 2.75 kW kg^−1^, respectively. All the above results demonstrate the feasibility of using sustainable nanocellulose as a nanoscale carbon substrate for the synthesis of hybrid composite electrodes toward renewable supercapacitor applications.

## 1. Introduction

Parallel to the growing need for renewable energy supply, there is an increasing demand for high-performance power sources with low-cost, lightweight, portable, and environmentally friendly features. Supercapacitors have emerged as a promising candidate for electrochemical energy storage because of their fast charge–discharge processes, providing a high energy density and a reasonable power density [1]. Both the materials and the structures are the cruxes in developing high-performance supercapacitors [2]. In terms of the materials, despite offering key performance advantages, many components are often expensive and contain fluorine, sulfur, and cyanide groups, which pose environmental hazards if discarded and disposed of using conventional landfill or incineration methods. Also, the derived supercapacitors sometimes suffer from a low energy density. Aiming at solving these problems, various categories of redox-reaction materials, including metal oxides, conductive polymers, and hybrid materials, have been employed as electrodes to dramatically improve the energy density. In regard to the structure, one key challenge is to achieve a high loading of electroactive materials per unit area with good contact of the electrodes. As the appeal of hybrid structures lies in the synergistic effects based on interfacial charge and energy transfer processes, controlling the nature of the interface and maximizing the interfacial area become critical issues. 

There is no doubt that hybrid structures of different nanomaterials would extend the diversity of micro- and macrostructures, with good integrated physical/chemical properties inherited from the constituent structures [3]. Manganese oxide (MnO*_x_*, i.e., Mn atoms with multiple oxidation states and phases) is an inexpensive pseudocapacitive material exhibiting high theoretical capacitance [4,5,6]. However, the small surface area and low conductivity of MnO*_x_* result in a poor charge−discharge rate for high-power applications. The combination of pseudocapacitive materials with carbon materials represents an important breakthrough for developing novel composite electrodes with improved performance [7]. 

Carbon materials are currently the most popular electrode materials used in supercapacitors. Among them, carbon nanotubes, graphene, and activated carbon have been investigated intensively in recent years [8,9,10]. However, the high cost of carbon nanomaterials and the complex preparation process (liquid phase deposition, electrodeposition, hydrothermal reaction, cyclic voltammetric anodic deposition, etc.) limit the fast development of carbon/MnO*_x_* electrode materials [11,12,13,14,15,16,17]. From both an availability and cost perspective, sustainable biochar and biochar nanomaterials are expected to be exploited to replace nonrenewable carbon materials [18,19,20,21,22]. Currently, activated carbons derived from biomass polymers have been identified as the most viable materials for supercapacitors [23,24,25]. A recent trend is the utilization of hierarchical porous graphitic carbons (HPGC), which combine the macroporous cores, mesoporous walls and micropores, as excellent support for metal oxides [26,27,28]. HPGC shows great potential in energy storage because of their high surface areas as well as short ion transport path [29,30]. However, most existing protocols rely heavily on nanocasting and soft templating, which usually use specific materials and thus make their industrial application unfeasible. Aiming at solving this problem, aerogels composed by nanocellulose have been considered as precursors of HPGC [31,32]. As a novel nanomaterial synthesized by biomass resources, nanocellulose was recognized as a sustainable building block for energy storage owning to its intrinsic structures and performance, as well as its renewable and abundant raw resources [33]. A large number of oxygen-containing functional groups on nanocellulose make them easier to decorate with electroactive materials and to modify the physicochemical properties [34,35,36,37,38,39]. Therefore, nanocellulose-based composite aerogel represents a versatile matrix to prepare lightweight hybrid materials [40,41,42,43,44]. Yang et al. have integrated spherical manganese dioxide nanoparticles and other kinds of active nanomaterials with the cellulose nanocrystal (a type of nanocellulose) aerogels, and lightweight, highly porous, and flexible hybrid aerogels for the development of supercapacitor materials [45]. Excellent capacitance retention at high charge–discharge rates was achieved due to the high mass ratio of active material to the total electrode mass, indicating the big potential of utilization of nanocellulose for high performance supercapacitors.

In this work, we aim to use forest waste-derived nanocellulose aerogel as the carbon nanomaterials and the nano-reservoirs for pseudocapacitive materials. Our nanocellulose exhibits high aspect ratio and can form strong entangled networks, resulting in self-standing and robust aerogels. Besides, the surface of nanocellulose contains large amounts of hydroxyl groups, which is good for the uniform integration of nanocellulose with the precursor of the active materials within the suspension. In addition, the nanocellulose composite aerogels can be effectively converted to carbon aerogels, which provides an efficient way for the integration of large amounts of electro-chemistry active materials into the carbon network to improve the ability for charge storage. The as-generated CNF/MnOx-based symmetric supercapacitor showed a satisfied energy density and power density. We hope our work can stimulate interest in the development of a “green” carbon source from biomass resources such as wood for advanced electrochemistry energy storage applications. 

## 2. Experimental Section

### 2.1. Preparation of Nanocellulose Suspension

Nanocellulose was extracted according to the method reported before [46]. Wood flour was first subjected to mild chemical pretreatment using acidified sodium chlorite and KOH to remove the lignin and hemicelluloses. After purification, the solution with 1 wt.% purified cellulose was ultrasonically disintegrated using a JY99-IIDN ultrasonicator (Scientz Biotechnology Co., Ningbo, China) at 1200 W for 20 min, resulting in a 1 wt.% nanocellulose suspension. 

### 2.2. Preparation of CNF/MnO_x_ Composite Aerogels

Typically, 0.1 g polyvinyl pyrrolidone (PVP) (Blue Season Technology Development Co. Ltd., Shanghai, China) and manganese acetate (Mn(OAc)_2_) (Komeo Chemical Reagent Development Center, Tianjin, China) (0.02, 0.06, 0.10, and 0.14 g) were added in a 20 g nanocellulose suspension and mechanically stirred for 1 h. The mixed solutions were frozen in a refrigerator at −18 °C for 24 h. Afterwards, they were subjected to freeze-drying using a Scientz-10N freeze dryer (Scientz Biotechnology Co., Ltd., Ningbo, China). The resultant hybrid aerogels were calcinated in a nitrogen atmosphere in an SK-G08123K tube furnace (Zhonghuan Experimental Furnace Co. Ltd., Tianjin China). The temperature rose from room temperature to 850 °C; the heating rate was 3 °C min^−1^. During the calcination process, the samples were kept at 270 °C for 1 h, 450 °C for 1 h, and 850 °C for 2 h, after the temperature dropped to room temperature. Finally, the CNF/MnO*_x_* composite aerogels were obtained. The composite aerogels were denoted as CNF/MnO*_x_*-*m*, where *m* takes on the value 1, 2, 3, or 4, respectively. Determined by the thermogravimetric analysis (TGA), the mass fraction of MnO*_x_* in the CNF/MnOx-*m* were calculated as ca. 6%, 18%, 30%, and 41%, respectively (Figure S1).

### 2.3. Preparation of Electrodes and Supercapacitors

The electrode was prepared by loading a slurry containing CNF/MnO*_x_*-*m*, poly(vinylidene fluoride) (PVDF) (in *N*-methylpyrrolidone), and acetylene black (8:1:1 wt.%) on a nickel collector. After the mixed materials were loaded, the electrode was pressed and dried in vacuum at 80 °C for 12 h. In the case of a three-electrode system, an Au-coated polyethylene terephthalate substrate was used as a working electrode, a mercurous sulfate electrode as the reference electrode, and Pt wire as the counter electrode. 1.0 M Na_2_SO_4_ solution was used as an electrolyte. A symmetric supercapacitor was assembled using two pieces of 1.5 × 1.5 cm CNF/MnO*_x_* electrodes (the average weight of each electrode was 2.0 mg), with 1.0 M LiPF_6_ ethylene carbonate/diethylene carbonate/ethyl-methyl carbonate (EC/DEC/EMC) (1:1:1 *v/v/v*) electrolyte-soaked separator in between. 

### 2.4. Characterizations

A JEM-2100 transmission electron microscope (TEM, JEOL Ltd., Tokyo, Japan) was used for the TEM imaging. Scanning electron microscopy (SEM) images were taken on a Quanta200 microscope (FEI, Hillsboro, OR, USA), at an operating voltage of 15 kV. The surface chemical composition was determined by an energy-dispersive X-ray (EDX) spectroscope connected to the SEM. Nitrogen adsorption–desorption isotherm measurements were performed using a 3H-2000PM2 Brunauer-Emmett-Teller (BET) surface area analyzer (Micromeritics, Beishide Instrument-ST Ltd., Beijing, China). The samples were degassed at 300 °C under vacuum for 5 h before the measurements. Specific surface area was calculated using the BET method. The total pore volume was determined at a relative pressure of 0.98. Pore volume and pore-size distribution were calculated using a nonlocal density functional theory (NLDFT) model based on the nitrogen adsorption branches of the isotherms. X-ray photoelectron spectroscopy (XPS) was performed using a K-Alpha system (Thermo Fisher Scientific, West Sussex, UK) operated at 14.0 kV and all binding energies were referenced to the C 1s neutral carbon peak at 284.8 eV. X-ray diffraction (XRD) patterns were obtained using a D/max 2200 X-ray diffractometer (Rigaku, Tokyo, Japan) with Ni-filtered CuK_α_ (λ = 1.5406 Å) radiation at 40 kV and 30 mA. Scattered radiation was detected in the range of 2*θ* = 10°−80° at a scan rate of 4° min^−1^. 

### 2.5. Electrochemical Measurements

Electrochemical measurements including cyclic voltammetry (CV) curves, galvanostatic charge–discharge (GCD) curves, and electrochemical impedance spectroscopy (EIS) were conducted on a CHI 660D electrochemical workstation (Chenhua Instrument Co., Shanghai, China) at room temperature. The electrochemical performance of individual electrodes was investigated using a three-electrode system prior to the fabrication of the supercapacitors. CV measurements were performed at different scan rates (2–100 mV s^−1^), within a potential window of –0.5 to 0.5 V. GCD measurements were performed with a constant current from 0.25–2 A g^−1^. EIS measurements were conducted for the working electrode in a frequency range of 100 kHz to 0.01 Hz with alternating current (AC) perturbation of 5 mV. The EIS data were analyzed using Nyquist plots, which represent the imaginary part (Z′) and the real part (Z″) of impedance. The performance of the supercapacitors was measured using a two-electrode method at scan rates of 5–100 mV s^−1^ within a potential window of 0 to 3.5 V.

The specific capacitance (*C*, F g^−1^) was calculated from the corresponding GCD curves at different current densities according to the equation:

(1) $$C=I/[(dV/dt)m]=\frac{I}{m}\frac{dt}{dV}$$

where *I*(A) is the response current, *m* is the average mass of the electrode, and *dt*/*dV* is the inverse of the slope of the discharge curve (V s^−1^).

The maximum specific energy density (*E*_max_) and power density (*P*_max_) were calculated according to the equations below:

(2) $$E_{\max}=0.5CV_{\max}^{2}/3.6$$

(3) $$P_{\max}=3600E_{\max}/t$$

## 3. Results and Discussion

**Figure 1:**
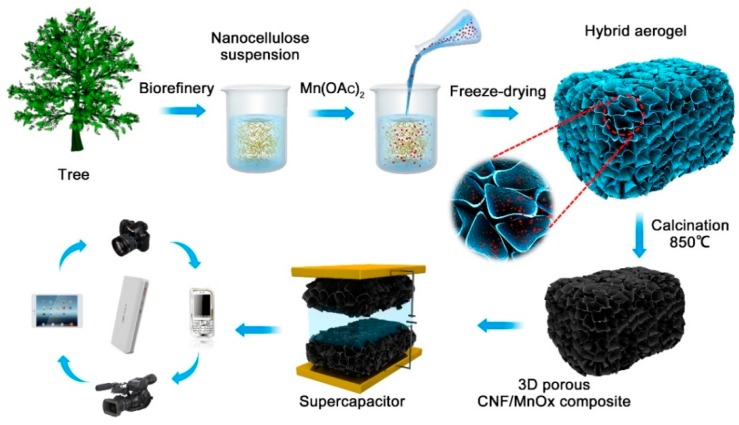
Schematic illustration of the preparation procedure of 3D porous carbonized nanocellulose fibers (CNF)/MnO*_x_* composite electrodes for supercapacitor applications.

**Figure 2:**
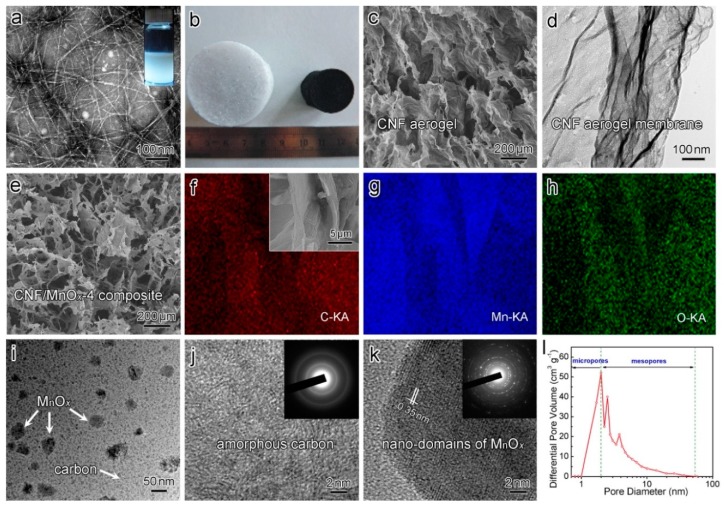
(**a**) Transmission electron microscope (TEM) image of nanocellulose fibers that extracted from wood biomass using a high-intensity ultrasonic nanofibrillation combined with chemical pretreatments. The inset in (**a**) shows the nanocellulose suspension. (**b**) Optical images of freeze-dried hybrid aerogel and the composite aerogel CNF/MnO*_x_*-4. (**c**,**d**) Scanning electron microscope (SEM) and TEM images exhibiting the 3D structure of CNF aerogel, which is made of carbon nanofiber membranes. (**e**) SEM image showing the 3D porous structure of CNF/MnO*_x_*-4. (**f**–**h**) SEM-Energy-dispersive X-ray (EDX) elemental mapping images show the electron probe microanalysis of C, Mn, and O elements, demonstrating the successful synthesis of MnO*_x_* nanoparticles on the CNF membranes. (**i**) TEM image showing the MnO*_x_* nanoparticles on CNF membranes. High-resolution TEM images showing (**j**) the amorphous carbon structure of CNF membranes, and (**k**) the crystalline nano-domains of MnO*_x_* nanoparticles that attached on the supporting CNF membranes. The inset in (**j**) shows the selected area electron diffraction (SAED) cyclic pattern of amorphous carbon structure. The inset in (**k**) shows the SAED multi-sets of hexagonal spots of MnO*_x_*. (**l**) Pore size distribution of CNF/MnO*_x_*-4.

The preparation procedure of the 3D porous CNF/MnO*_x_* composite electrodes for supercapacitors is illustrated in Figure 1. First, nanocellulose fibers were disintegrated from wood waste through biorefinery steps. The resultant nanocelluloses exhibited fibrous structures with 2–20 nm widths and lengths exceeding 1 μm (Figure 2a) [47]. Because of high aspect ratios and numerous hydroxyl groups being exposed, the nanocellulose fibers are likely to intertwine together, which facilitates the formation of the gel network, particularly for mass concentrations above 0.8 wt.% [48]. Subsequently, the nanocellulose suspension was mixed with Mn(OAc)_2_ solutions containing different concentrations of solute, and freeze-dried into freestanding hybrid aerogels with tailored shapes and sizes. The obtained hybrid aerogels showed no significant differences in appearance compared with the nanocellulose aerogels. After calcination at 850 °C in an argon atmosphere, the hybrid aerogels turned into CNF/MnO*_x_*-*m* composite aerogels, and the colors changed into black (Figure 2b). The shape of the composites was maintained but the volume shrunk by almost 40%. Thus, the bulk density increased from 0.01 g cm^−3^ to 0.16 g cm^−3^. Meanwhile, the composites exhibited flexible and elastic features. 

SEM and high-resolution TEM were applied to investigate the microstructure of the CNF/MnO*_x_* composite aerogels. The SEM images of the CNF aerogel in Figure 2c,d show the 3D porous structure made of carbon nanofiber membranes. The CNF/MnO*_x_* composite aerogels also exhibit a similar 3D network structure (Figure 2e). The SEM-EDX elemental mapping images of C, Mn, and O atoms evidently display the homogeneous distribution of MnO*_x_* nanoparticles on the surfaces of the CNF membranes (Figure 2f–h). The high-resolution TEM image and the selected area electron diffraction (SAED) cyclic pattern in Figure 2j further reveal the graphite carbon structure of the CNF membranes. The MnO*_x_* nanoparticles that attached to the CNF membranes possess typical crystalline nanodomains (Figure 2k). The CNF aerogel has a high BET surface area (*S*_BET_) value of 554.8 m^2^ g^−1^, whereas that of CNF/MnO*_x_*-4 drops to 219.3 m^2^ g^−1^. The pore sizes of CNF/MnO*_x_*-4 were calculated 1.99−305.3 nm from the pore-size distribution curves. The mesopore (2.0−58.0 nm) fraction and micropore (≤1.95 nm) fraction were calculated as 80.25% and 31.50%, respectively (Figure 2l). The unique structural characteristics of the composite are expected to benefit the absorption of electrolytes and provide diffusion channels for electrolyte ions. 

**Figure 3:**
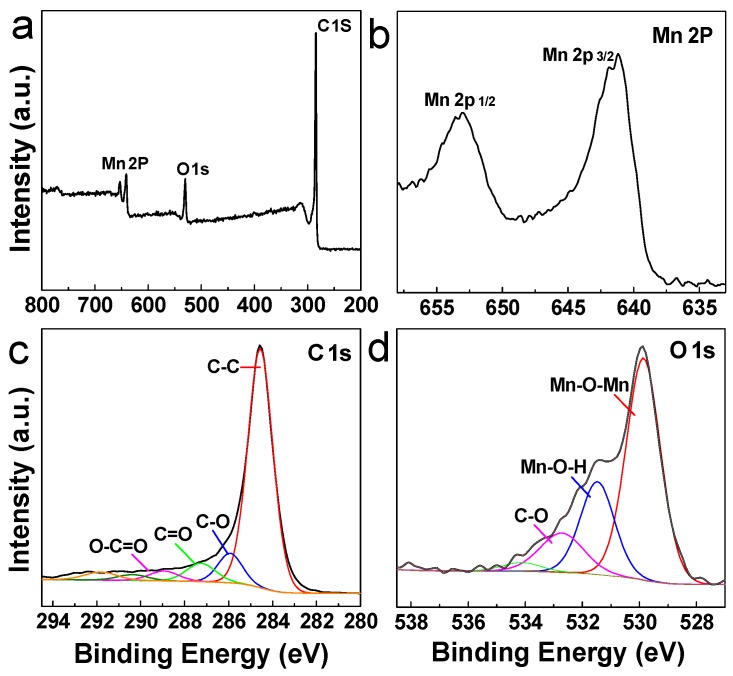
(**a**) X-ray photoelectron spectroscopy (XPS) survey spectrum of CNF/MnO*_x_*-4. (**b**–**d**) XPS high-resolution spectra of Mn 2p, C 1s, and O 1s.

The XPS survey was performed to determine the surface chemical state of the CNF/MnO*_x_* composite aerogels, and the spectra of the CNF/MnO*_x_*-4 are shown as representative examples. In the wide-scan spectrum (Figure 3a), the composition elements only contain C, O, and Mn, whereby the content of C is higher than the others, suggesting deep graphitization of CNF. This is also demonstrated by the fitting curve of the C 1s profile that is related to C–C bonds (sp^2^ carbon) at a chemical shift of 284.5 eV (Figure 3c). Mn can assume multiple oxidation states (Mn^2+^, Mn^3+^, and Mn^4+^) and diverse phases during both oxidation and reduction processes. There are two peaks of Mn 2p_1/2_ and Mn 2p_3/2_, and the energy difference between these two peaks is 11.6 eV (Figure 3b). According to the fitting curves of the O 1s profile (Figure 3d), the three deconvoluted peaks can be assigned to the chemical bonds of Mn–O–Mn, Mn–O–H, and C–O at binding energies of 529.9, 531.4, and 532.7 eV, respectively. All these indicate that two kinds of manganese oxides are generated during the calcination process. The formation of a mixed-valent compound rather than single-phase MnO*_x_* is attributed to oxygen deficiency during the calcination process.

**Figure 4:**
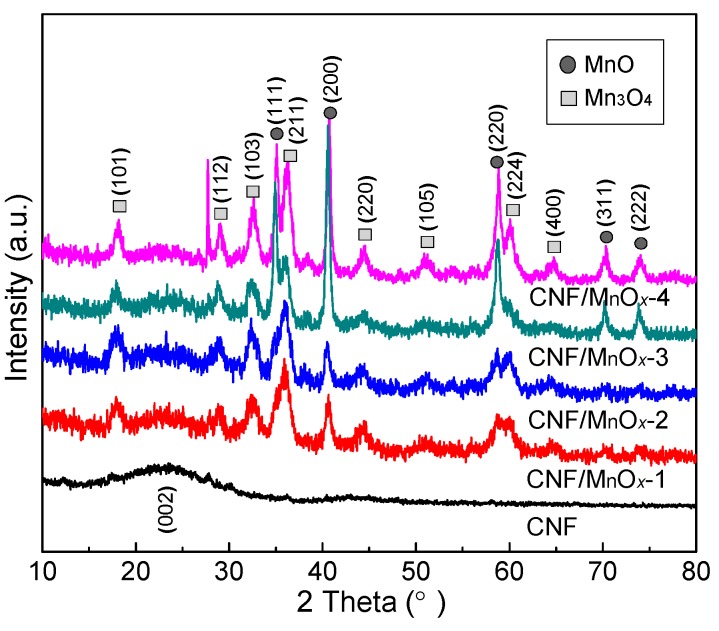
X-ray diffraction (XRD) patterns of CNF and CNF/MnO*_x_* composite aerogels.

The phase structure of the CNF/MnO*_x_* composite aerogels was further analyzed from a crystallographic perspective. Figure 4 shows the relevant XRD patterns. The wide amorphous peak in the range of 2*θ* = 18–30° is ascribed to CNF. A small diffraction peak is observed at 2*θ* = 23.5°, which may be ascribed to the (002) lattice plane of highly graphitized carbon. The mixed crystals of Mn_3_O_4_ and MnO can be identified from the diffraction curves of the CNF/MnO*_x_* composites. The diffraction peaks (101), (112), (103), (211), (220), (224), and (400) at 2*θ* = 18.02°, 28.96°, 32.42°, 36.04°, 44.36°, 60.02°, and 64.66°, respectively, agree well with the Mn_3_O_4_ (faceted single crystals) powder standard (JCPDS 18-0803). The appearance of diffraction peaks at (111), (200), (220), (311), and (222) at 2*θ* = 34.90°, 40.54°, 58.72°, 70.18°, and 73.80°, respectively, agrees well with the MnO powder standard (JCPDS 07-0230). A comparison of the peak intensities at 34.90°, 40.54°, and 58.72° that correspond to the lattice planes (111), (200), and (220), respectively, suggests that the appearance of a MnO phase was related to an increase of Mn(OAc)_2_ content. The limited supply of oxygen molecules cannot afford the oxidization of total Mn^2+^ to Mn^3+^. Thus, both the XRD patterns of CNF/MnO*_x_*-3 and CNF/MnO*_x_*-4 display the characteristic diffraction peaks of MnO and Mn_3_O_4_. Mixed-valent MnO*_x_* usually contains both donor and acceptor sites in its microstructures as well as defects and mismatch induced by different phases, which can enable a high charge-storage capacity. 

**Figure 5:**
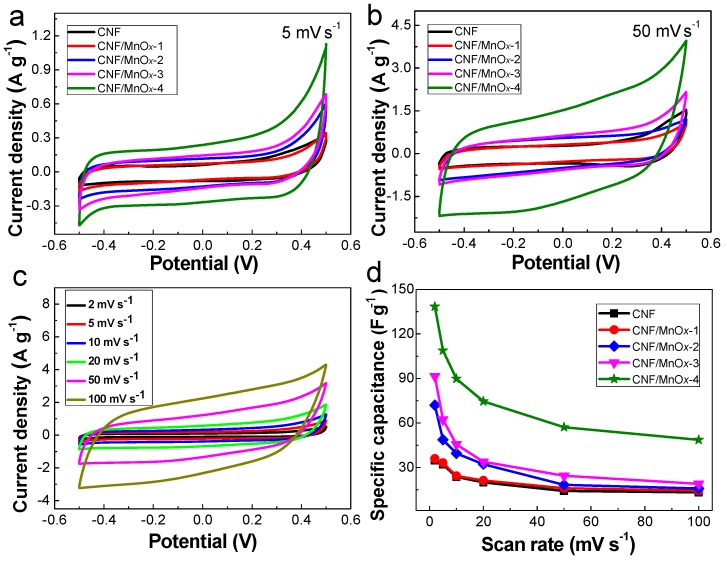
Cyclic voltammetry (CV) curves of the CNF/MnO*_x_* electrodes at a scan rate of (**a**) 5 mV s^−1^ and (**b**) 50 mV s^−1^. (**c**) Changes of CV curves of CNF/MnO*_x_*-4 at scan rates from 2 to 100 mV s^−1^. (**d**) Specific capacitance vs. scan rate.

The electrochemical performance of the CNF/MnO*_x_* electrodes was evaluated from the CV measurements. All the CNF/MnO*_x_* composites exhibit symmetric quasi-rectangular CV shapes, indicating a high reversible charge-storage capacity (Figure 5a). This is attributed to the successive multiple surface redox reactions of MnO*_x_*, behaving with a charge-storage mechanism that is different from that of most metal oxides. No obvious oxidation and deoxidization peaks are observed, but orientation polarization is displayed. The hysteresis at a relative pressure between 0.4 and 0.5 confirms the existence of mesopores. At a high scan rate of 50 mV s^−1^, the CNF/MnO*_x_* electrodes also exhibit quasi-rectangular CV shapes, and the symmetry is improved (Figure 5b). As the scan rate is increased from 2 to 100 mV s^−1^, the response current improves and shows certain multiplying power characteristic, and the orientation polarization subsides (Figure 5c). The amount of MnO*_x_* also has a significant influence on the specific capacitance of CNF/MnO*_x_* composites (Figure 5d). The specific capacitance of CNF/MnO*_x_*-1 is 35.9 F g^−1^, which is similar to that of pure CNF (34.8 F g^−1^). The specific capacitances of CNF/MnO*_x_*-2 and CNF/MnO*_x_*-3 are twice and three times that of pure CNF. Notably, CNF/MnO*_x_*-4 shows the highest specific capacitance (138.4 F g^−1^). The reduction of specific capacitance at high scan rates is assumed to be caused by the limiting redox reaction of MnO*_x_* and hysteresis of the charge–transfer coefficient. When the mass fraction of MnO*_x_* was further increased, the specific capacitance started to decrease (Figure S2).

**Figure 6:**
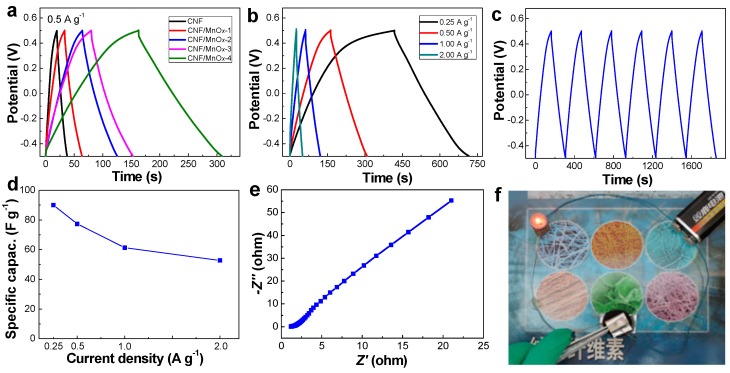
Galvanostatic charge–discharge (GCD) curves of the electrode made of CNF/MnO*_x_* electrodes. (**a**) Charge–discharge curves of CNF/MnO*_x_* electrodes in a three-electrode configuration measured at a constant current of 0.5 A g^−1^. (**b**) Charge–discharge curves of CNF/MnO*_x_*-4 electrode at different current densities. (**c**) GCD curves of CNF/MnOx-4 electrode at a constant current of 0.5 A g^−1^. (**d**) Specific capacitance vs. current density of the CNF/MnO*_x_*-4 electrode. (**e**) Nyquist plot for CNF/MnO*_x_*-4 electrode. (**f**) Optical image shows the electroconductivity of CNF/MnO*_x_*-4 electrode in a serial circuit.

The CNF/MnO*_x_* electrodes were evaluated by means of GCD measurements within −0.5 to 0.5 V. The charge–discharge curves exhibit an almost triangular shape with a small internal resistance drop (Figure 6a). The deviation of the charge–discharge curves from the linear voltage–time relationship indicates a pseudocapacitive behavior. In aqueous electrolytes, the pseudo-capacitance of MnO*_x_* comes from the intercalation and de-intercalation of protons and/or alkali ions, as well as redox reactions. The ion intercalation and de-intercalation and redox reactions take place on the surface of the MnO*_x_* under high-speed charge–discharge. The presence of the internal resistance drop at the beginning of the discharge is usually associated with the equivalent series resistance phenomenon. At the same current density, the CNF/MnO*_x_*-4 electrode shows superior performance to the others. The discharge curves of the CNF/MnO*_x_*-4 electrode display a nearly linear response at different current densities from 0.25 to 2.0 A g^−1^ with a corresponding discharge time of 300 s and 30 s, respectively (Figure 6b). Figure 6c shows a typical GCD curve of a CNF/MnO*_x_*-4 electrode under continuous operation for the first several cycles, which indicates ideal electrochemical reversibility and almost symmetric charge–discharge properties. The specific capacitance can be calculated from the discharge curves using Equation (1) and shows a downward tendency with the rise of current density (Figure 6d). The Nyquist plot displays a typical diffusion-controlled Warburg capacitive behavior with a diagonal line in the low-frequency region and a small depressed semicircle in the high-frequency region. From the EIS results, the Nyquist plot shows an intercept at the real impedance (*Z*′) of 1.1 Ω (Figure 6e). An elongated ‘semicircle’ can be attributed to the widely distributed pore sizes in the structure. The relatively small serial resistance suggests a compact electrode system that ensures fast charge transfer between electrolyte and electrodes. Figure 6f shows good electroconductivity of CNF/MnO*_x_*-4 electrode in a series circuit. From above all, CNF/MnO*_x_*-4 was selected as the optimal sample for constructing a symmetric supercapacitor.

**Figure 7:**
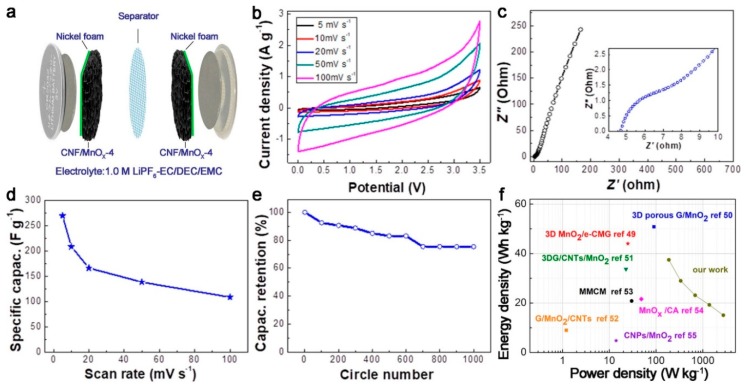
Electrochemical measurement of the CNF/MnO*_x_*-4 based symmetric supercapacitor: (**a**) Schematic illustration of the CNF/MnO*_x_*-4-based symmetric supercapacitor; (**b**) CV curves obtained at different scan rates; (**c**) Nyquist plots; (**d**) Specific capacitance vs. scan rate; (**e**) Cyclic stability at a charge–discharge current density of 2 A g^−1^ for 1000 cycles; (**f**) Ragone plot.

Figure 7a is the schematic illustration of the CNF/MnO*_x_*-4-based symmetric supercapacitor. Figure 7b shows a series of rate-dependent CV curves of the CNF/MnO*_x_*-4-based symmetric supercapacitor in the voltage range of 0–3.5 V. The CV measurements were conducted at a scan rate of 5, 10, 20, 50, and 100 mV s^−1^. Similar to the results measured in a three-electrode configuration, the assembled supercapacitor exhibited quasi-rectangular CV curves. Moreover, even at a high scan rate of 100 mV s^−1^, the CV curves remain symmetrical, suggesting good reversibility of the electrochemical processes. From the EIS results, the Nyquist plot shows an intercept at *Z*′ of 4.6 Ω (Figure 7c). Furthermore, the low-frequency region of the impedance curves shows a nearly straight sloping line, suggesting a good capacitive performance of the electrode. The calculated specific capacitances at different scan rates are present in Figure 7d. The CNF/MnO*_x_*-4-based symmetric supercapacitor shows specific capacitances from 269.7 F g^−1^ to 108.6 F g^−1^ as the scan rate rises from 5 to 100 mV s^−1^. The specific capacitances represent an 80% retention of the initial capacitance after 1000 cycles of charging–discharging at 2 A g^−1^ (Figure 7e). 

As noted from the CV and GCD studies, high energy density and power density are expected for such a supercapacitor assembly. Energy density and power density of the CNF/MnO*_x_*-based symmetric supercapacitor are calculated from the GCD profiles and are illustrated in a Ragone plot (Figure 7f). A maximum energy density of 37.5 Wh kg^−1^ can be stored at a power density of 2.75 kW kg^−1^, under the same charge–discharge condition at a voltage of 3.5 V. From the Ragone plot, the performance of our supercapacitor can compare with or superior to those of CNT/MnO_2_-based supercapacitors and graphene/MnO_2_-based supercapacitors [49,50,51,52,53,54,55] (e.g., 3D MnO_2_/grapheme [49], graphene/CNT/MnO_2_ [51], graphene/MnO_2_/CNT supercapacitors [52]).

The satisfied performance of our supercapacitor is mainly contributed to the synergistic effects of material chemistry and structure on the electrochemistry performance of CNF/MnO*_x_*-based electrodes, which are summarized and ascribed in the following aspects: (1) The mixture of nanocellulose suspension and Mn(OAc)_2_ solution is uniform and homogeneous, which prevents the aggregation of MnO*_x_* colloids. (2) The CNF membranes are fabricated as the carbon aerogel carriers for MnO*_x_* and nanoreservoirs for electrolytes. (3) A large number of research data have concluded that the electrical properties of carbon/MnO*_x_* composite depend on the mass fraction of MnO*_x_*. When the mass fraction of MnO*_x_* is less than 70%, the electrical properties increase with the increase of the mass of MnO*_x_*. In this experiment, the optimal MnO_x_ loading is just half of the best, indicating a high utilization efficiency of MnO_x_. (4) The web-like conductive percolating structure facilitates the diffusion path of electrolytes and reduces the charge-transfer impedance. Electrons can easily travel through the diffusion path and can be picked up by MnO_4_^−^ ions at other locations. (5) The high electrical conductivity and low charge-transfer resistance improve the kinetics significantly and are favorable to overcome the kinetic limitations at high-current operation. (6) The integrated structure contains the essential components (electrodes, spacer, and electrolyte) of an electrochemical device, paving the way for designing a variety of energy-storage devices [43].

## 4. Conclusions

Freestanding CNF/MnO*_x_* composite aerogels were facilely synthesized by freeze-drying an aqueous dispersion of nanocellulose and Mn(OAc)_2_, followed by a calcination process. Despite the straightforward and inexpensive preparation, the shaped composites showed 3D porous structures with hierarchical pores, which benefit the transportation of both electrolyte ions and electrons. The composites exhibit quasi-rectangular CV curves, pseudocapacitive behavior, and small impedance. When used in a supercapacitor, the as-prepared CNF/MnO*_x_*-based symmetric supercapacitor showed a specific capacitance of 269.7 F g^−1^ and possessed an energy density as high as 37.5 Wh kg^−1^, as well as a power density of 2.75 kW kg^−1^. Meanwhile, the supercapacitor displayed cyclic stability with capacitance retention of 80% after 1000 charge−discharge cycles at 2 A g^−1^. The approach is therefore highly promising in terms of both cost efficiency and scale-up for the development of renewable energy-storage devices toward sustainability.

## Supplementary Materials

The following are available online at http://www.mdpi.com/2073-4360/11/1/129/s1, Figure S1: TGA curves of CNF/MnO*_x_* composite aerogels, Figure S2: CV curves of the CNF/MnO*_x_* electrodes at scan rate of (a) 5 mV s^−1^ and (b) 50 mV s^−1^.
