# Supplementary material for: Composite Aerogels of Carbon Nanocellulose Fibers and Mixed-Valent Manganese Oxides as Renewable Supercapacitor Electrodes
Source: Polymers (Basel). 2019 Jan 13;11(1):129. doi: 10.3390/polym11010129 (PMC6404137; doi:10.3390/polym11010129)
Supplement: Supplementary file 1 [file polymers-11-00129-s001.pdf]

Supporting Information

Composite Aerogels of Carbon Nanocellulose Fibers and Mixed-Valent Manganese Oxides as Renewable Supercapacitor Electrodes

Xiaoyu Guo[†], Qi Zhang[†], Qing Li[†,*], Haipeng Yu[†,*], Yixing Liu[†,*]

† Key laboratory of Bio-based Material Science and Technology, Ministry of Education, Northeast Forestry University, Harbin 150040, China.

* E-mail: liqing007007@126.com; yxl200488@sina.com; yuhaipeng20000@nefu.edu.cn

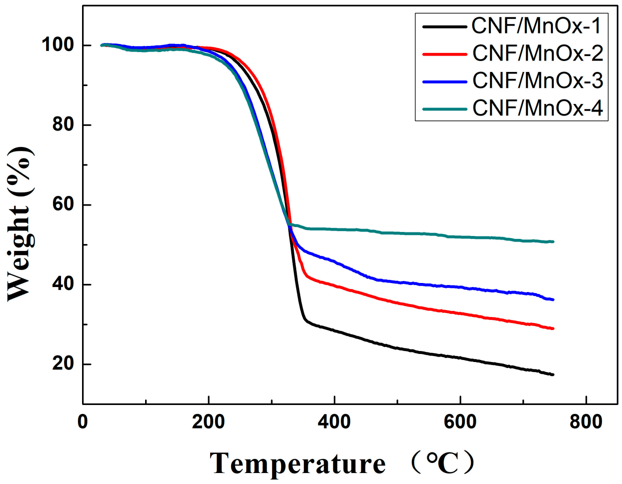

Figure S1 TGA curves of carbonized nanocellulose fibers (CNF)/MnO_x composite aerogels. The following test data are the TG test curves of samples heated from room temperature to 800 °C (heating rate is 3 °C min^{-1}).

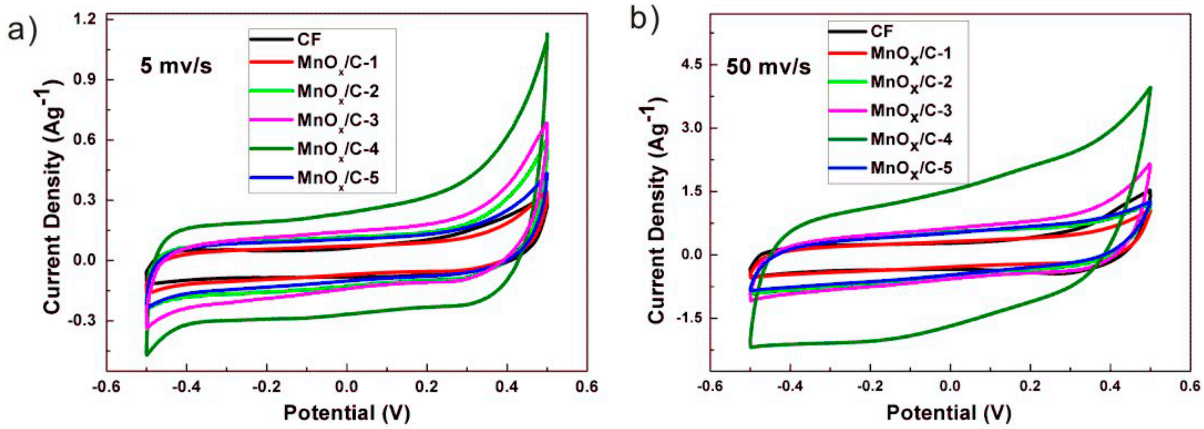

Figure S2 Cyclic voltammetry (CV) curves of the CNF/MnOx electrodes at scan rate of (a) 5 mV s^{-1} and (b) 50 mV s^{-1}. Typically, 0.1 g PVP, $Mn(OAc)_2$ (0.02, 0.06, 0.10, 0.14 g, and 0.20g) were added in a 20g nanocellulose suspension, the composite aerogels were denoted as CNF/MnO_x-*m*, where *m* takes the value of 1, 2, 3, 4, or 5.
